# Error in Author Byline Credential

**DOI:** 10.1001/jamanetworkopen.2022.21224

**Published:** 2022-07-07

In the Original Investigation titled “Concordance of Clinician-Documented and Imaging Response in Patients With Stage IV Non–Small Cell Lung Cancer Treated With First-Line Therapy”^1^ published on May 12, 2022, there was a missing PhD credential in the byline for Dr Bourla. This article has been corrected.^1^
